# Quantifying Explainability in OCT Segmentation of Macular Holes and Cysts: A SHAP-Based Coverage and Factor Contribution Analysis

**DOI:** 10.3390/diagnostics16010097

**Published:** 2025-12-27

**Authors:** İlknur Tuncer Fırat, Murat Fırat, Taner Tuncer

**Affiliations:** 1 Faculty of Medicine, Inonu University, Malatya 44050, Turkey; ilknur.tuncer@inonu.edu.tr; 2 Faculty of Medicine, Malatya Turgut Ozal University, Malatya 44900, Turkey; murat.firat@ozal.edu.tr; 3 Department of Computer Engineering, Firat University, Elazığ 23200, Turkey

**Keywords:** macular hole, optical coherence tomography, OCT, deep learning, segmentation, explainable artificial intelligence, SHAP analysis

## Abstract

**Background**: Optical coherence tomography (OCT) can quantify the morphology and dimensions of a macular hole for diagnosis and treatment planning. Objective: The aim of this study was to perform automatic segmentation of macular holes (MHs) and cysts from OCT macular volumes using a deep learning-based model and to quantitatively evaluate decision reliability using the model’s focus regions and GradientSHAP-based explainability. **Methods**: In this study, we automatically segmented MHs and cysts in OCT images from the open-access OIMHS dataset. The dataset comprises 125 eyes from 119 patients and 3859 OCT B-scans. OCT B-scan slices were input to a UNet-48-based model with a 2.5D stacking strategy. Performance was evaluated using Dice and intersection-over-union (IoU), boundary accuracy was evaluated using the 95th-percentile Hausdorff distance (HD95), and calibration was evaluated using the expected calibration error (ECE). Explainability was quantified from GradientSHAP maps using lesion coverage and spatial focus metrics: Attribution Precision in Lesion (APILτ), which is the proportion of attributions (SHAP contributions) falling inside the lesion; Attribution Recall in Lesion (ARILτ), which is the proportion of the true lesion covered by the attributions; and leakage (Leakτ = 1 − APILτ), which is the proportion of attributions falling outside the lesion. Spatial focus was monitored using the center-of-mass distance (COM-dist), which is the Euclidean distance between the attribution center and the segmentation center. All metrics were calculated using the top τ% of the pixels with the highest SHAP values. SHAP features were clustered using PCA and k-means. Explanations were calculated using the clinical mask in ground truth (GT) mode and the model segmentation in prediction (Pred) mode. **Results**: The Dice/IoU values for holes and cysts were 0.94/0.91 and 0.87/0.81, respectively. Across lesion classes, HD95 = 6 px and ECE = 0.008, indicating good boundary accuracy and calibration. In GT mode (τ = 20), three regimes were observed: (i) retina-dominant: high ARIL (hole: 0.659; cyst: 0.654), high Leak (hole: 0.983; cyst: 0.988), and low COM-dist (hole: 7.84 px; cyst: 6.91 px), with the focus lying within the retina and largely confined to the retinal tissue; (ii) peri-lesional: highest ARIL (hole: 0.684; cyst: 0.719), relatively lower Leak (hole: 0.917; cyst: 0.940), and medium/high COM-dist (hole: 16.22 px; cyst: 10.17 px), with the focus located around the lesion; (iii) narrow-coverage: primarily seen for cysts in GT mode (ARIL: 0.494; Leak: 1.000; COM-dist: 52.02 px), with markedly reduced coverage. In Pred mode, the ARIL20 for holes increased in the retina-dominant cluster (0.758) and COM-dist decreased (6.24 px), indicating better agreement with the model segmentation. **Conclusions**: The model exhibited high accuracy and good calibration for MH and cyst segmentation in OCT images. Quantitative characterization of SHAP validated the model results. In the clinic, peri-lesion and narrow-coverage conditions are the key situations that require careful interpretation.

## 1. Introduction

A macular hole is a condition that, in many cases, develops following vitreofoveal traction within the vitreoretinal interface (VMI) spectrum. The clinical picture is characterized by full- or partial-thickness foveal tissue loss, leading to a decrease in central visual acuity. With appropriate surgery, anatomic closure rates are high and are often accompanied by functional recovery [1,2]. Delay in diagnosis and treatment can result in chronic holes and a worse visual prognosis. Therefore, early diagnosis and timely surgical planning are crucial for anatomic closure and visual outcomes [1,2]. Optical coherence tomography (OCT) is the gold standard for the diagnosis and classification of VMI diseases. The OCT-based anatomic classification of the International Vitreomacular Traction Study (IVTS) Group defines vitreomacular adhesion (VMA), vitreomacular traction (VMT), and macular holes based on criteria such as hole size and the presence of VMT. This framework directly contributes to clinical management and prognosis prediction [3,4]. Furthermore, OCT is indispensable for treatment planning and follow-up [3,4,5].

Automated analysis of OCT images of the VMI spectrum using deep learning (DL) is becoming increasingly common. Recently, there have been many improvements in the diagnostic accuracy and workflow efficiency of classification/segmentation approaches for OCT images [6,7]. Open data initiatives have accelerated method development by providing labeled data for multiple retinal pathologies, including in the VMI [8]. Herath et al. compared U-Net variants for MH segmentation and achieved high and similar performance across models when evaluated based on Dice and HD95 [9]. Hu et al. predicted postoperative closure status using multicenter data and a DL-based model and reported good discrimination and clinical applicability [10]. Lachance et al. successfully predicted visual acuity recovery using a hybrid approach combining B-scan DL features and clinical variables [11]. Mikhail et al. systematically summarized the current evidence and future clinical use of artificial intelligence in the management of MHs in a review [12]. Kwon et al. predicted postoperative macular anatomy with a conditional VAE-based generative model. They reported ≥85% accuracy and layer agreement between artificial intelligence-assisted optical coherence tomography (AI-OCT) and ground truth optical coherence tomography (GT-OCT) for restoration of the external limiting membrane (ELM) and ellipsoid zone (EZ) [13]. Recent generative validation studies indicate that long-term OCT prediction is feasible in eye diseases, including 12-month forecasts in AMD and for postoperative macular hole anatomy [13,14].

The 2.5D approach adds subtle volumetric context to each slice by combining consecutive adjacent B-scans within a limited window, providing a practical balance between the limited context of 2D and the memory and labeling costs of 3D approaches [6,7]. The proposed model uses the 2.5D-UNet-48 architecture for automatic segmentation of macular holes and cysts from OCT macular volumes. In addition to standard performance metrics, it evaluates SHAP-based explanations via quantitative comparison with masks and interprets distinct SHAP regimes through factor analysis. The main objectives of this study can be summarized as follows:

- To achieve a balance between the limited context of 2D and the memory and labeling costs of 3D approaches using a 2.5D approach that integrates adjacent slice information at a low cost.
- To propose a computationally efficient UNet-48 segmentation backbone (48→384 channel hierarchy) with Group Normalization, enabling full-resolution (512 × 512) OCT segmentation under limited GPU memory and stable training with small mini-batches.
- To quantify the in-lesion share of SHAP attributions, their leakage beyond the lesion, and the focus–center distance by comparing GradientSHAP maps with masks, and to summarize how these patterns align with the lesion via factor analysis.
- To translate this SHAP analysis into a clinical tool that provides case-level confidence/warning signals, and to clarify how and why results may be misleading, and to facilitate validation of automated measurements with explanations.
- To automate the morphological detection of macular holes and cysts, providing decision support for surgical planning and follow-up with well-calibrated probabilities.
- To demonstrate the computational efficiency of the 2.5D architecture and its potential for integration into clinical workflows through anatomical post-processing.

## 2. Materials and Methods

### 2.1. Dataset

**Figure 1:**
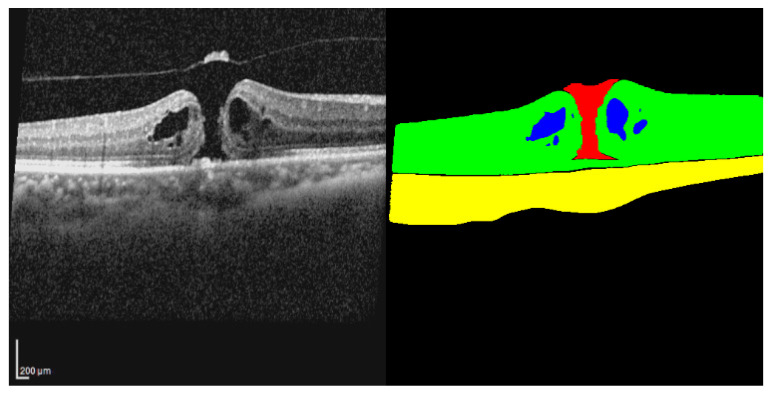
An example OCT–mask pair from the dataset. The raw OCT image is shown on the left, and the corresponding mask is shown on the right. The mask shows hole in red, cyst in blue, retina in green, choroid in yellow, and background in black.

This study used the open-access OIMHS dataset [15,16]. Since the OIMHS data are anonymized and publicly available, no additional ethical approval was required. Data collection was conducted with approval from Zhejiang Provincial People’s Hospital (QT2023024) [16]. The dataset contains OCT B-scans of full-thickness macular hole (FTMH) cases and corresponding segmentation masks for each slice. The masks are labeled with four classes: retina, macular hole, intraretinal cyst, and choroid. Thus, each raw OCT slice is paired with a mask image of the same size and resolution. In addition to the OCT B-scans, the dataset also contains information on image-quality and participant demographics. In our study, these masks were directly used for training, testing, and evaluation. An image–mask pair from the dataset is presented in Figure 1.

The dataset consists of 125 eyes from 119 patients and 3859 OCT B-scans. The mean age was 64.1 ± 11.6 years; 74.8% of patients were female (*n* = 89) and 25.2% were male (*n* = 30). Among the eyes that were imaged, 44.8% were right eyes (*n* = 56) and 55.2% were left eyes (*n* = 69). The hole stage distribution was as follows: 0.8% stage 1 (*n* = 1); 12.8% stage 2 (*n* = 16); 27.2% stage 3 (*n* = 34); and 59.2 stage 4 (*n* = 74).

### 2.2. Method

**Figure 2:**
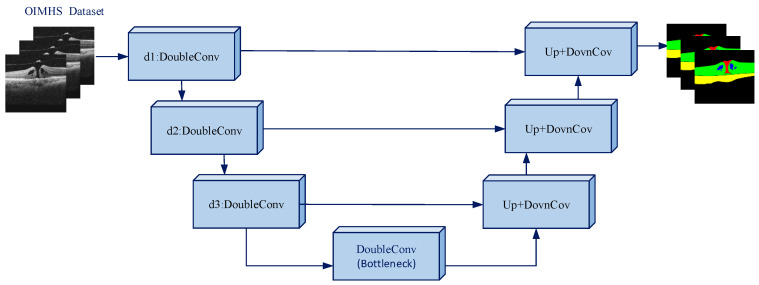
Model structure. The schematic shows a 2.5D U-Net-like symmetric encoder–decoder architecture. The input is downsampled by three DoubleConv blocks (d1–d3), with a Bottleneck at the center. The output is upsampled by three Up + DownConv blocks, combining skip connections with encoder features to preserve both detail and context.

U-Net, a type of convolutional neural network (CNN), has demonstrated strong segmentation performance on biomedical images. The main idea is to downsample the spatial dimensions of the feature map to retain salient features while discarding less useful information, and then learn compact representations at a bottleneck layer and upsample back to the original resolution. The structure of the proposed model is shown in Figure 2.

Raw OCT B-scan images and the corresponding segmentation masks were organized on a per-eye basis. Masks were converted to pixel-level class identities using robust color matching against a fixed color palette for the retina, hole, cyst, and choroid classes; the background was assigned to black. For the model input, a 2.5D stacking strategy was used: each center slice was combined with a window of three consecutive slices, and reflective padding was applied to avoid boundary effects. All slices were rescaled to 512 × 512 pixels and normalized using channel-based z-score standardization. Light quality-control steps were performed to improve data quality; degraded or saturated slices were eliminated, and visual verification was performed on a small sample of each subset.

Before splitting, eye IDs were matched to hole stage, and eyes were divided in a stage-balanced manner into 70% training/15% validation/15% test. Splitting was performed on a per-patient basis; all eyes from the same patient and all slices from each eye were assigned to a single subset.

A UNet-48-based segmentation model was designed using 2.5D packed OCT slices as input. The network has an encoder-decoder structure, with convolutional layers and Group Normalization units used in each block. Group Normalization was selected because it is more stable than Batch Normalization with small mini-batches. The encoder extracts a feature hierarchy with increasingly smaller spatial resolutions, while the decoder symmetrically amplifies this information to generate the segmentation mask.

The model input is an image $x\in R^{B\times C\times H\times W}$ with height *H*, width *W*, batch size *B*, and channel count *C* (*C* = 3; images are 3 × 512 × 512). For a 5-class segmentation, the base channel counts in the encoder, decoder, and output are as follows:

Encoder: 48→96→192→384;

Decoder: 384→192→96→48;

Output: 48→5 (1 × 1 Conv).

Table 1 summarizes layer details.

The model outputs, for each pixel i, a class-probability vector $p_{i}=(p_{i,1},\ldots ,p_{i,c})$. Here, C represents the number of classes (5: retina, hole, cyst, choroid, and background), and $p_{i,c}$ represents the probability that pixel i belongs to class *c*. During training, a combination of Dice loss and Focal–Tversky loss is used to update the model parameters. These losses complement each other in addressing class imbalance and improving the capture of small lesions. The Dice coefficient measures the overlap between the predicted and ground truth masks (Equation (1)).

(1) $$Dice\left(c\right)=\frac{2\sum_{i}y_{i,c}\;p_{i,c}}{\sum_{i}y_{i,c}+\sum_{i}p_{i,c}+\varepsilon }$$

where $y_{i,c}\in \left\{0,1\right\}$ indicates whether pixel i belongs to class *c*, and *ε* is a small constant for numerical stability. The Dice loss is defined in Equation (2).

(2) $$L_{Dice}=1-\frac{1}{C}\sum_{c=1}^{C}Dice(c)$$

The Tversky index (Equation (3)) is defined by two weights that control the balance of false positives and false negatives. In this study, α = 0.7 was used for false positives and *β* = 0.3 for false negatives, with *ε* added for numerical stability:

(3) $$TI\left(c\right)=\frac{\sum_{i}y_{i,c}\;p_{i,c}}{\sum_{i}y_{i,c}p_{i,c}+\alpha \sum_{i}(1-y_{i,c)}p_{i,c}+\beta \sum_{i}y_{i,c}\left(1-p_{i,c}\right)+\varepsilon }$$

The Focal–Tversky loss is written in exponential form to increase the sensitivity to small/difficult samples; the exponent γfocal was used to control the focal effect (*γfocal* = 1.5) (Equation (4)):

(4) $$L_{FT}=\frac{1}{C}\sum_{c=1}^{C}{\left(1-TI\left(c\right)\right)}^{\gamma focal}$$

Here, *γfocal* > 1 gives more weight to errors in small and difficult lesions. The total loss is the weighted combination of Dice and Focal–Tversky losses (Equation (5)):

(5) $$L_{Total}=\lambda L_{Dice}+\left(1-\lambda \right)L_{FT},\;\lambda =\frac{2}{3}$$

## 3. Results

In this study, we propose a U-Net-based system for automatic segmentation of macular holes (MHs) and cysts in OCT images. The experimental setup procedure for the proposed model is detailed below. The hardware and software environments used for the experiments are summarized in Table 2.

Given our 2.5D input setting (*C* = 3 stacked adjacent slices) at a 512 × 512 resolution and our hardware constraints (RTX 4050 Laptop GPU with 6 GB VRAM), we adopted a UNet-48 backbone (48→96→192→384) to balance the representational capacity and memory footprint. Wider backbones or full 3D modeling would increase activation memory and force smaller batches or downsampling. Since we trained with small mini-batches (batch size: 3), we used Group Normalization (8 groups), which is more stable than BatchNorm under small-batch regimes.

To mitigate class imbalance, class weights were added (larger weights for small-volume classes and smaller weights for large-volume classes). Slight augmentations were applied to increase data diversity: z-jitter (*p* = 0.20), channel dropout (*p* = 0.10), low-level speckle noise (*σ* = 0.02, *p* = 0.50), and small-scale affine transforms (rotation ≤ 5°, translation ≤ 3%, scale 0.97–1.03, and shear ≤ 1.5°). Horizontal flip was used with *p* = 0.50. Training used AdamW (initial learning rate: 1.5 × 10^−4^; weight decay: 1 × 10^−2^). The learning rate followed a cosine annealing schedule with an initial ~800-step warm-up. For numerical stability, the upper bound of the gradient clipping was set to 1.0. Training was planned for a maximum of 24 epochs (mini-batch size: 3). Early stopping was performed on the validation set by monitoring the Dice (hole) metric: warm-up (8 epochs), patience (5 epochs), and minimum recovery threshold Δ = 0.002. Training stopped when improvement fell below this threshold.

Horizontal flip Test-Time Augmentation (TTA) was applied to each slice to average the probabilities of the original and projected outputs of the model; mixed precision was used for speed/stability when possible.

Lesion predictions were trimmed to anatomical boundaries with an intraretinal constraint. Small noise islands were removed by applying opening + closing for holes and closing for cysts. Neighborhood-slice consistency (±1 slice) was enforced by majority rule, and fragments below size thresholds proportional to the image area were suppressed. Slice and eye-level masks were generated. Performance is reported as macro Dice/IoU. HD95 was calculated for boundary quality, and ECE for calibration. Representative overlays and summary tables were generated.

SHAP attributes each input feature a signed contribution based on game-theoretic Shapley values, providing additive, pixel-level attributions for model predictions [17]. We used GradientSHAP because it combines SmoothGrad-style noise smoothing with Integrated Gradients’ path integration to approximate Shapley values under input perturbations; compared with Grad-CAM (coarser, layer-level maps) and Vanilla Integrated Gradients (baseline/saturation sensitivity), GradientSHAP provides more stable, fine-grained attributions that align better with segmentation masks and support our quantitative metrics. To manage the computational cost of dense, pixel-level attributions, we performed GradientSHAP offline on a pre-selected subset of lesion-positive target slices (*n* = 370 in this study), rather than on all slices. Target slices were chosen from the evaluation split by only retaining those with non-empty hole/cyst masks (in GT and/or prediction mode, depending on the analysis mode), and attribution metrics were only computed for the relevant class/mode pairs. Inputs were standardized with channel-wise z-score. We computed GradientSHAP attributions by averaging gradients over 128 samples/16 baselines. Baselines were smoothed using a slight Gaussian blur (*σ* = 0.8); positive contributions were normalized, and percentile clipping (0.1–99.9) was applied. To ensure anatomical consistency, the heatmap was displayed only within the retina; attributions outside the retina were suppressed. This suppression was applied only for visualization clarity and did not affect the model outputs. Full-field heatmaps can be generated by disabling the retinal masking when needed. As the reference distribution, we employed Gaussian fuzzy noise, which preserves global intensity statistics while attenuating edges. This avoids artificial contours from zero/black baselines in OCT images and reduces edge-contrast bias, producing smoother, less spurious attributions near anatomical boundaries.

Heatmaps were ranked by attribution magnitude and binarized to obtain top-10% (τ = 0.10) and top-20% (τ = 0.20) masks, allowing for the evaluation of narrow-focus (τ = 0.10) and wider-focus (τ = 0.20) regimes.

All metrics were calculated separately using two reference masks: (i) Ground truth (GT)-referenced mode: SHAP attributions and model output were compared with the GT mask; coverage, leakage, and focus-consistency were measured with respect to GT lesion boundaries. (ii) Prediction (Pred)-referenced mode: The same metrics were calculated based on the model’s own segmentation mask, assessing the self-consistency of the explanations with the model’s decisions. This dual view allowed us to assess the consistency of the explanations with both the clinical labels and the model’s own decisions. The SHAP targets and running parameters are summarized in Table 3.

The following metrics were used: segmentation quality (Dice, IoU, HD95, and ECE) in the validation and testing phases, and explanation quality (APILτ, ARILτ, Diceτ, Leakτ, COM-dist, and nCOM) in the SHAP analysis phase, each computed in the ground truth (GT) and prediction (Pred) modes. In this study, APILτ, ARILτ, Diceτ, and Leakτ were calculated for τ = 10 and 20; τ = 5 was also used in the factor analysis section. APIL10 denotes APILτ with τ = 10. We performed a sensitivity analysis over τ ∈ {5%, 10%, 20%} (top-τ% attribution thresholds) and report the full τ-sweep results in Supplementary Tables S2–S4. Our main SHAP-based conclusions were consistent across τ levels.

Present-only metrics were computed only on slices that contain a lesion. For each class, Dice and IoU were computed per slice and then averaged across slices (macro per-slice). Consequently, the averaged Dice and IoU are not expected to satisfy the closed-form conversion IoU = Dice/(2 − Dice). Unless otherwise stated, metrics included absent-class slices, which contribute a neutral value due to smoothing; ‘present-only’ averaging was used only in the analyses explicitly marked using this method. All explanation metrics are reported separately for the GT (clinical label alignment) and Pred (model self-consistency) modes. The scope and usage of parameters are summarized in Table 4.

To make SHAP visualizations more interpretable, we applied factor analysis. In addition to the explanation metrics, we defined Leak20 (=1 − APIL20, proportion of attribution outside the lesion), Lesion Pixels (LP) (lesion area), Positive Attribution Mass (PAM) (total positive SHAP contribution), and Focus Concentration (FC = APIL5/APIL20). To control for size effects, we used $nCOM=nCOM-dist/\sqrt{Lesion\;area}$ and its sign-reversed form Focus Proximity (FP = −nCOM); a higher FP indicates that attributions are concentrated closer to the lesion.

All features were scaled with StandardScaler, followed by a three-component principal component analysis (PCA). We then extracted three clusters with k-means in the PC1–PC2 space. The outputs include loading heatmaps (PC1–PC3), a PC1–PC2 scatterplot, and cluster-wise metric means. The component interpretations were as follows: (i) PC1 captures the success–leakage contrast, which increases when APIL and Dice load positively and Leak20 loads negatively, corresponding to lesion-focused, low peri-lesion patterns; (ii) PC2 reflects retinal containment and spatial focusing, which rises with ARIL and FP, indicating contributions concentrated within the retina near the lesion; (iii) PC3 emphasizes size-independent focusing, which is driven mainly by FP.

k-means clustering revealed three regimes in the PC1–PC2 plane: (i) a retina-dominant regime—where the focus is mainly within the retina with limited lesion overlap (low APIL/Dice, and high Leak20); (ii) a peri-lesional regime—where the focus extends around the lesion (low APIL and Dice are relatively higher; Leak20 is relatively lower but still noticeable); and (iii) a narrow-coverage regime—where APIL and Dice are near zero, Leak is near one, and the intraretinal coverage is low. Cluster names are data-driven and chosen for visual interpretation; each represents a deviation from an ideal intra-lesion focus. This holistic view combines multiple metrics in a low-dimensional space to jointly examine focus–coverage–leakage dynamics. Loadings summarize metric–component relationships, while scatter/cluster plots show how the samples are distributed across regimes. For clarity, Algorithm 1 summarizes the overall end-to-end workflow of the proposed segmentation and regime-based quantitative XAI pipeline.
**Algorithm 1.** Overview of the proposed pipeline1.  Split patients/eyes into train/val/test (70/15/15, leakage-safe).2.  Pack inputs as 2.5D (C = 3) or 2D (C = 1).3.  Split patients/eyes into train/val/test (70/15/15, leakage-safe).4.  Evaluate on test set and report Dice/IoU (and HD95/ECE).5.  Select lesion-positive target slices for XAI analysis (GT- and Pred-referenced).6.  Compute GradientSHAP attribution maps per class (offline).7.  Threshold at top-τ% (τ ∈ {5, 10, 20}) and compute APILτ, ARILτ, Diceτ, Leakτ, and COM-dist.8.  Assign samples to regimes (retina-dominant/peri-lesion/narrow-coverage) and summarize results.

SHAP summaries were cross-tabulated with image-quality flags (signal shield, image blur, and low signal strength) and all analyses followed the present-only rule. Unless otherwise noted, comparisons used τ = 20 (Dice20, APIL20, and Leak20). Cyst size was stratified by area quartiles; small cysts were defined as the lower quartile (Q1) with a threshold of 518 pixels and compared with medium/large cysts; Cohen’s d was reported where applicable. For image-quality flags (signal shield and image blur), we report Δ = flagged − clean differences. The best/worst slices were selected by macro-Dice. For each sample, we show the mask, Top-10 Attribution–Lesion Overlay (T10 overlay), and SHAP heatmap together. Finally, success quartiles Q1–Q4 were derived from present-only Dice20, reporting the per-quartile metric means and counts of hole-present/cyst-present slices.

**Figure 3:**
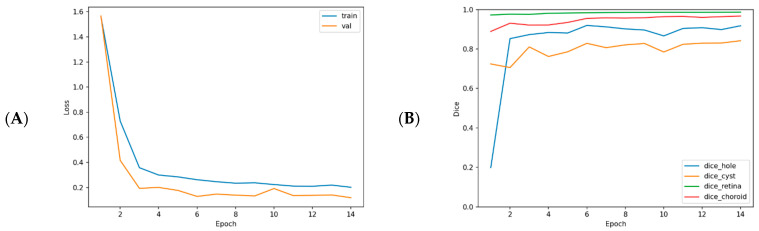
Training/validation loss curves and the change in Dice metrics by class and epoch during the training process. (**A**) Both curves plateau after a rapid decrease; the small gap between curves suggests no clear overfitting. (**B**) While retina and choroid segmentations stabilize with high accuracy in the early period, a slower but steady increase is observed in the hole and cyst classes. This demonstrates that the model struggles with small and irregular lesions but performs well in overall accuracy.

Figure 3 presents the training and validation curves and the per-class Dice evolution across epochs.

While the UNet-48/GN (512 × 512) model achieved high Dice/IoU values in the retina, hole, and choroid classes on the test set, the cyst scores were lower. The lesion-class HD95 mean was 6.0 px, indicating small boundary errors. The ECE was 0.008 (computed on hole/cyst only slices), suggesting good calibration (Table 5). To contextualize these results, we evaluated classic U-Net baselines (UNet-64 + BatchNorm) at full resolution (512 × 512) on the same test split in both 2D (*C* = 1) and 2.5D (*C* = 3) settings, alongside our proposed 2.5D UNet-48 + GroupNorm. The proposed model achieved a Dice/IoU of 0.947/0.915 (mean, BG included), with a macular hole Dice/IoU of 0.941/0.910 and cyst Dice/IoU of 0.874/0.805. In comparison, the 2D U-Net baseline reached a Dice/IoU of 0.920/0.874, with a macular hole Dice/IoU of 0.917/0.883 and cyst Dice/IoU of 0.843/0.771, while the 2.5D U-Net-64 baseline obtained an overall Dice/IoU of 0.877/0.836 and macular hole Dice/IoU of 0.709/0.708. These results support our choice of UNet-48 as a more memory-efficient backbone for 2.5D segmentation under limited GPU resources and Group Normalization as a stable normalization choice for small mini-batches (see Supplementary Table S1).

**Figure 4:**
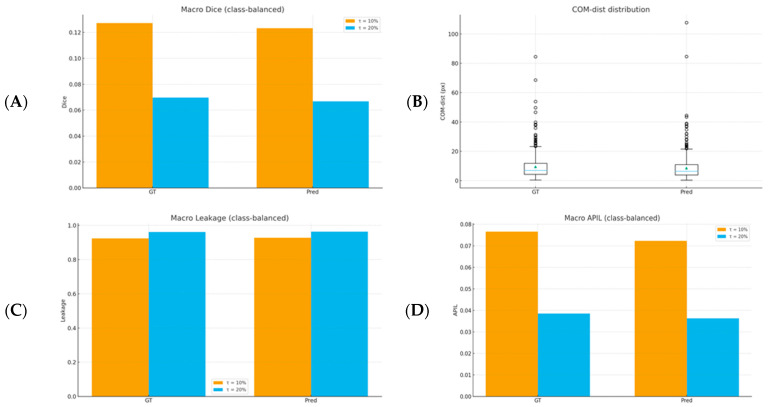
Summary of class-balanced SHAP metrics. (**A**) Macro Dice (τ = 10% & τ = 20%; GT vs. Pred), (**B**) COM-dist distribution (GT vs. Pred), (**C**) macro leakage (τ = 10% & τ = 20%), and (**D**) macro APIL (τ = 10% & τ = 20%).

The macro scores in Pred mode were below those in GT, indicating better alignment of annotations with clinical labels (GT) and a more conservative focus compared to model segmentation. In terms of spatial accuracy, COM-dist was lower in Pred mode (8.98 px vs. 10.24 px), meaning the SHAP center of mass lay closer to the lesion center in Pred than in GT, suggesting a stronger fit (Table 6). As τ increased from 10% to 20%, the top-τ area increased, while Dice and APIL decreased and Leak increased; COM-dist remained lower in Pred mode (Figure 4).

For holes, the peri-lesion (*n* = 79) cluster in GT mode showed the highest intraretinal coverage (ARIL20 = 0.684) with relatively lower leakage (Leak20 = 0.917). Meanwhile, the COM-dist of 16.22 px and FP of −0.21 indicate a shift in focus to the peri-lesion region; the large area (LP = 6487.4 px) supports this pattern. In the retina-dominant cluster (*n* = 77), ARIL20 = 0.659, but APIL20/Dice20 (0.017/0.032) was low and Leak20 was high (0.983); however, the COM-dist of 7.84 px and FP of −0.24 indicate a closer focus. The LP of 1282.4 px indicates that the field is smaller and the FC of 3.99 indicates a tight focus. Two main improvements were evident in Pred mode: (i) ARIL20 increased in both main clusters, and (ii) spatial proximity improved. Furthermore, a narrow-coverage cluster (*n* = 15) appeared, representing difficult slices with small/partial holes where coverage failed. The cluster-level FP, LP, PAM, and FC indicators add an operational layer to clinical interpretation by combining focal proximity, lesion size, total positive contribution, and focal tightness (Table 7).

For cysts, in GT mode, the peri-lesion (*n* = 113) cluster reflected a peripheral focus in large lesions (LP = 4386.4 px) with an ARIL20 of 0.719, Leak20 of 0.940, COM-dist of 10.17 px, and FP of −0.16. The retina-dominant (*n* = 253) cluster showed a relatively close but non-specific focus near the lesion, with an ARIL20 of 0.654, very low APIL20/Dice20 (0.012/0.024), Leak20 of 0.988, COM-dist of 6.91 px, and FP of −0.49. The narrow-coverage cluster (*n* = 4) was very small (LP = 7.0 px) and represented coverage failure (Leak20 = 1.00, ARIL20 = 0.494, FP = −22.59, and COM-dist = 52.02 px). In Pred mode, ARIL20 increased significantly in all main clusters, and spatial proximity improved; partial recovery was also observed in the narrow-coverage cases. Overall, LP was largest in PL clusters, while FP/COM-dist (focal proximity), PAM (positive contribution), and FC (focal tightness) provide complementary cues for clinical interpretation (Table 8).

The narrow-coverage regime was markedly less prevalent in both modes (hole: 0% in GT and 9.3% in Pred; cyst: 1.1% in GT and 3.9% in Pred). In cysts, the retinal-dominant cluster was approximately twice as common as the peri-lesional cluster (GT: 68.4% vs. 30.5%; Pred: 65.0% vs. 31.1%), while for holes, the two patterns were similarly frequent (GT: 49.4% vs. 50.6%; Pred: 46.6% vs. 44.1%). The higher narrow-coverage rate in Pred than GT (hole: 0% vs. 9.3%; cyst: 1.1% vs. 3.9%) suggests that the model produced narrow-coverage foci more often when explaining its own segmentations. These may signal false-positive bias or partial/incomplete coverage that warrant extra validation. By contrast, the retina-dominant/peri-lesion distributions were closely matched across modes, indicating that the primary pattern mix was preserved; the difference stemmed mainly from the rise in the narrow-coverage cluster.

**Figure 5:**
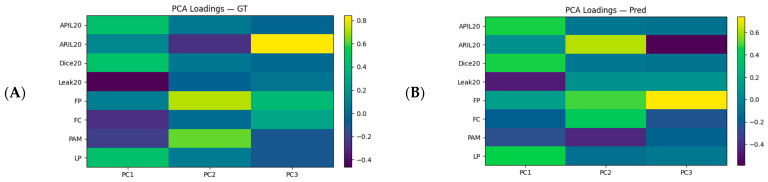
PCA loading maps: (**A**) GT; (**B**) Pred. The color scale represents the loading coefficient (−0.4…+0.8); warm tones indicate positive loadings, and cool tones indicate negative loadings. As the absolute loading magnitude increases (≈0.4 and above), the corresponding metric more strongly defines that component.

Three components emerged from the factor analysis: PC1 represents the contrast between focus/coverage and leakage (positive APIL and DICE, negative Leak); PC2 captures intraretinal localization and spatial proximity (ARIL and FP positive); and PC3 reflects pure focus without size effects (FP dominant). The GT and Pred patterns were similar. Loadings were more pronounced in Pred, particularly in PC3, suggesting a relatively stronger representation of the focus component in the model descriptions (Figure 5).

**Figure 6:**
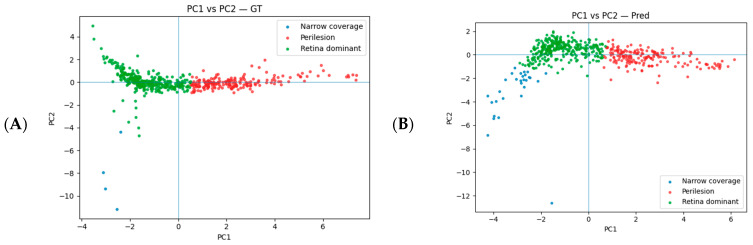
PC1–PC2 distribution and annotation regimes: (**A**) GT; (**B**) Pred. The upper-right region corresponds to good coverage and low Leak with intraretinal, near-lesion focus; the lower-left region corresponds to poor coverage and extra-retinal/far focus. Moving right on the horizontal axis, APIL/Dice increase and Leak decreases; moving up on the vertical axis, ARIL and FP increase.

On the PC1–PC2 plane, the samples split into three regimes: retinal-dominant (high ARIL, limited APIL/Dice, high Leak, and generally low–moderate FP), peri-lesion (high Leak and relatively better APIL/Dice), and narrow-coverage (APIL/Dice ≈ 0, low ARIL, Leak ≈ 1, and very negative FP). In Pred, the clusters separated more clearly, indicating stronger self-consistency of the explanations with the model’s segmentation. Clinically, the peri-lesion regime can shift heat toward bright band/layer edges; narrow-coverage regimes often reflect incomplete coverage in small or fragmented cysts; and retinal-dominant concentrates within the retina have limited lesion overlap (Figure 6).

**Figure 7:**
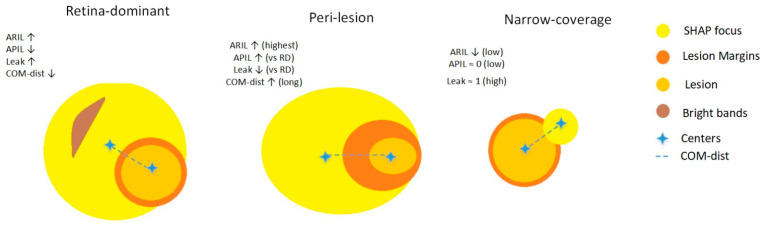
Schematic representation of three SHAP focus profiles. Retina-dominant (**left**): yellow focus mostly within the retina near the lesion with limited lesion overlap; COM-dist short, ARIL high, APIL low, Leak high; the brown band at the border suggests a possible false-positive cue. Peri-lesion (**center**): yellow focus ring-shaped around the lesion; COM-dist increases, APIL increases, and Leak decreases relative to retina-dominant; the orange arc marks prominent lesion margins. Narrow-coverage (**right**): yellow focus confined to a small area; ARIL/APIL low, Leak high, COM-dist moderate/variable.

The SHAP maps were divided into three patterns: (i) retina-dominant patterns where the heat primarily concentrates within the retina near the lesion. The spatial focus is consistent but the lesion specificity is limited (low APIL/Dice and high Leak). ARIL is high (though lower than in peri-lesion patterns), and COM-dist is generally short. This regime is typically driven by intra-retinal reflectivity—non-specific reflectance and layer-texture inside the retina—so high-contrast yet non-diagnostic cues attract attributions to nearby retinal tissue without precisely overlapping the lesion. (ii) In peri-lesion patterns, heat concentrates around the lesion perimeter and along layer edges, and partly penetrates into the lesion. As a result, ARIL is highest, APIL/Dice are higher, and Leak is lower than in retinal-dominant patterns, while COM-dist is longer (the ring shifts the focus outward). This regime is typically driven by lesion-border hyperreflectivity—sharp, reflective rims (bright bands/specular reflections) and boundary artifacts—which pull attributions to the rim and can be amplified by mild over-segmentation across the boundary. (iii) In narrow-coverage patterns, heat captures only part of the lesion, particularly in small/fragmented cysts or thick retinal sections. Here, APIL/Dice are very low (≈0), ARIL is low, Leak ≈ 1, and COM-dist is variable, indicating incomplete coverage. Accordingly, a retinal-dominant pattern can be characterized as “retina-focused but non-specific,” a peri-lesion pattern can be characterized as having “marginal/artifact risk,” and narrow-coverage patterns have “small-lesion sensitivity” (Figure 7).

In GT mode, peri-lesion cases showed the strongest overlap scores (e.g., hole Dice/IoU = 0.842/0.737, cyst Dice/IoU = 0.840/0.747), while retina-dominant cases were slightly lower but comparable (hole: 0.831/0.736; cyst: 0.805/0.714). In cysts in GT mode, narrow-coverage corresponds to reduced overlap (0.776/0.647, with a small sample count), consistent with difficult small/fragmented lesions. In Pred mode, we observe the same overall tendency that peri-lesion remains strong (hole 0.847/0.744, cyst 0.842/0.749), supporting the use of narrow-coverage and/or attribution drift as a practical ‘review-needed’ flag. We summarize the segmentation quality (Dice/IoU) stratified by SHAP regime in Supplementary Tables S5 and S6 to support the intended use of regimes as reliability/warning indicators.

**Figure 8:**
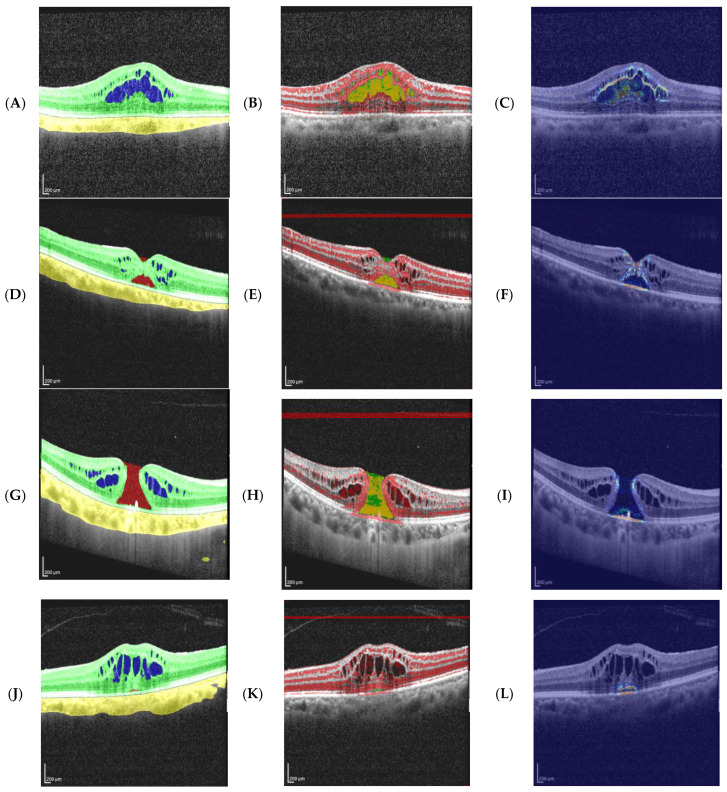
Representative SHAP examples (OCT + mask on the left, T10 overlay in the middle, heatmap on the right). In the overlay, green = lesion mask, red = top-10% positive SHAP, and yellow = intersection. In the heatmap, warmer colors indicate stronger positive contribution. Case metrics are reported for τ = 10 and τ = 20 (px = pixels). (**A**–**C**) (Retinal-dominant, GT—cyst): APIL20 = 0.102, Dice20 = 0.181, Leak20 = 0.898; APIL10 = 0.200, Dice10 = 0.318, Leak10 = 0.800; COM-dist = 2.85 px. Contribution is concentrated near the lesion inner rim within the retina; the cyst nucleus shows weaker response. (**D**–**F**) (Retinal-dominant, Pred—hole): APIL20 = 0.021, Dice20 = 0.042, Leak20 = 0.979; APIL10 = 0.043, Dice10 = 0.081, Leak10 = 0.957; COM-dist = 10.45 px. Partial coverage with marked leakage; sensitivity to RPE border cues. (**G**–**I**) (Peri-lesion, Pred—hole): APIL20 = 0.075, Dice20 = 0.136, Leak20 = 0.925; APIL10 = 0.150, Dice10 = 0.248, Leak10 = 0.850; COM-dist = 21.94 px. Extralesional spread with poor centration (focus shifted toward the rim). (**J**–**L**) (Narrow-coverage, Pred—hole): APIL20 = 0.00008, Dice20 = 0.00015, Leak20 = 0.99992; APIL10 = 0.00015, Dice10 = 0.00030, Leak10 = 0.99985; COM-dist = 2.32 px. Focus lies close to the lesion but does not penetrate it; the hole is very small and there is no intersection (yellow).

Figure 8 shows the typical focus patterns of the retinal-dominant (RD), peri-lesion (PL), and narrow-coverage (NC) profiles in real slices. Focus centering, leakage, and coverage patterns varied across clusters, consistent with the APIL, Dice, Leak, and COM-dist values.

In the best–worst eye comparison, the Dice score for holes was stable with an approximately 3% difference between the best and worst eyes, whereas cyst Dice decreased by approximately 13%. The performance loss was therefore primarily driven by cysts. In small cysts (*n* = 252), Dice = 0.784 ± 0.202; in medium/large cysts (*n* = 118), it was 0.906 ± 0.036, yielding Δ = −0.122 (Cohen’s d = −0.73).

ECE was low for both classes (hole: 0.007; cyst: 0.010). Based on performance quartiles, there were fewer slices with holes (*n* = 7) and slices with cysts were more common (*n* = 90) in the lowest-performing group; in the highest-performing group, the number of slices with holes increased (*n* = 53) while the number of slices with cysts was similar (*n* = 89). This suggests that low-performing samples were cyst-dominant and harder cases, whereas high-performing samples showed more stable overall segmentation.

**Figure 9:**
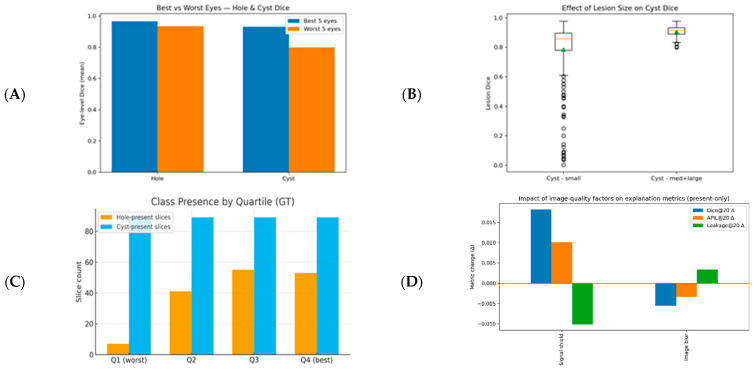
(**A**) Hole/cyst Dice comparison in the best vs. worst 5 eyes: hole is stable (~3% difference), decrease is mainly due to cyst (~13%). (**B**) Size effect: performance is significantly lower in small cysts (Q1, area < 518 px). (**C**) Performance quartiles (present-only Dice20): Q1 cyst-heavy (hole = 7, cyst = 90), Q4 is more balanced (hole = 53, cyst = 89). (**D**) Image quality (present-only, Δ = flagged − clean): signal shield +[Dice20, APIL20]/−[Leak20]; image blur −[Dice20, APIL20]/+[Leak20].

For slices flagged with signal shield (*n* = 47), we observed a Dice20 Δ = +0.018, APIL20 Δ = +0.010, and Leak20 Δ = −0.010; for image blur (*n* = 12), Dice20 Δ = −0.0056, APIL20 Δ = −0.0034, and Leak20 Δ = +0.0034. Signal shield appears to direct attention into the lesion (higher focus/coverage, lower leakage), whereas image blur introduces edge ambiguity (lower focus/coverage, higher leakage). Δ is not reported for low signal strength because there were no positive examples (Figure 9).

**Figure 10:**
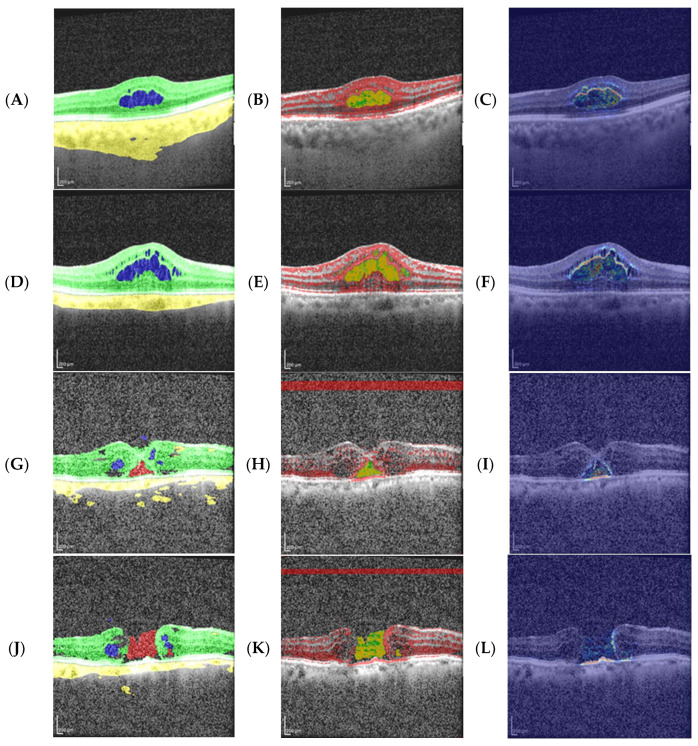
Representative best–worst examples (OCT + mask on the left, T10 overlay in the middle, heatmap on the right). (**A**–**C**) Best 1: Large, well-circumscribed cyst—high accuracy and intralesional focus (Macro Dice = 0.984; APIL20 = 0.063, Dice20 = 0.117, Leak20 = 0.090; nCOM = 0.199). (**D**–**F**) Best 2: similar pattern; good coverage/focus, low leakage (Macro Dice = 0.979; APIL20 = 0.107, Dice20 = 0.189, Leak20 = 0.0893; nCOM = 0.097). (**G**–**I**) Worst 1: small/fragmented cyst with an accompanying hole—poor coverage, very high leakage (APIL20 = 0.023/0.021; Dice20 = 0.044/0.040; Leak20 = 0.977/0.979; nCOM = 0.243). (**J**–**L**) Worst 2: medium cyst with a large hole—limited coverage, significant leakage (APIL20 = 0.029/0.067; Dice20 = 0.057/0.122; Leak20 = 0.97/0.93; nCOM = 0.900). In summary, as lesion size and artifact load increase, the focus shifts outside the lesion (e.g., retinal border/RPE line); in good samples, the heat is localized within the lesion.

In the best examples, the cysts are large and well-defined, segmentation was high, and annotation was focused within the lesion. In the worst examples, small/fragmented cysts and focal distortion due to layer-boundary artifacts were predominant. This contrast is consistent with the size effect and trends in the quadrant analysis (Figure 10).

## 4. Discussion

In our study, we achieved a Dice/IoU of 0.941/0.910 for macular hole (MH) segmentation and Dice/IoU of 0.874/0.805 for cyst segmentation on the OIMHS dataset. Using the same dataset, Herath et al. reported the best MH performance with InceptionNetV4 + U-Net (Dice = 0.9672) and emphasized that HD95 may be unreliable for small structures, highlighting the importance of overlap-based metrics for MH evaluation [9]. Kulyabin et al. also used the same OIMHS benchmark and reported overall Dice scores of 0.913 (MH) and 0.902 (IRC) using prompt-driven volumetric segmentation with SAM 2/MedSAM 2, highlighting strong performance under a different interaction/evaluation setting than our fully automatic pipeline [18]. Although the OIMHS dataset is a public benchmark, direct numerical comparisons across studies should be interpreted cautiously because train/validation/test splits, preprocessing choices, and interaction/evaluation settings may differ. For reproducibility, we used a stage-balanced, patient-level 70/15/15 split (keeping all slices from the same patient/eye in a single subset) and the report results on the held-out test set. Using a different dataset, Frawley et al. demonstrated that volumetric 3D U-Net variants can achieve strong MH overlap (mean IoU = 0.876 ± 0.012, with reported example Dice values in the 0.94–0.97 range) [19]. Automated 3D segmentation has also been applied to MH morphology and measurements, reporting high validation accuracy (99.19%) and clinically meaningful variability in anatomical measurements [20]. Similarly, Pereira et al. showed that automated MH volume estimation can closely match manual grading (R^2^ = 0.94) and may correlate with postoperative visual recovery better than the minimum linear diameter [21]. For intraretinal cyst segmentation, prior work reports lower overlap scores than for MH segmentation, with Dice = 0.71 in Girish et al. [22], mean Dice = 0.78 (OPTIMA)/0.81 (KERMANY) in Ganjee et al. [23], and Dice = 0.69 (OCSC)/0.67 (DME)/0.79 (AEI) in Gopinath and Sivaswamy [24], underscoring the higher difficulty of cyst delineation. Overall, Table 9 places our results within the context of recent literature and suggests that our 2.5D UNet-48 design provides competitive segmentation accuracy while remaining computationally practical.

The 2.5D U-Net offers a practical balance between the context-free nature of 2D approaches and the memory/labeling costs of 3D approaches: lower hardware requirements and shorter training times [19,25,26], consistent segmentation with multiple scan planes and strong data augmentation [27], and reduced boundary ambiguity by incorporating neighboring-slice context [28,29,30]. The computational efficiency and accuracy trade-offs of lightweight (MobileNetV2 + U-Net) and heavyweight (InceptionV4 + U-Net) backbones have been demonstrated using the OIMHS dataset [9]. Recent OCT studies also report strong results with alternative paradigms. Kulyabin et al. demonstrate that prompt-driven foundation models can achieve high volumetric biomarker segmentation performance on the OIMHS dataset under an interaction-based evaluation setup, which is not directly comparable to fully automatic 2D/2.5D/3D baselines [18]. In contrast, Toğaçar et al. proposed a CNN-activation–based retinal disease detection approach and reported overall accuracies of 99.60%, 99.89%, and 97.49% across three OCT datasets, highlighting the broader interest in activation/attribution-driven interpretability beyond segmentation [31]. Our aim was not only to achieve high accuracy but also to leverage the advantages of the 2.5D approach to transform saliency annotations into regime-based confidence signals and integrate them into clinical practice. To this end, we quantified the annotations using SHAP coverage metrics and factor analysis.

Accurate, qualitative, and quantitative identification of macular hole morphology is critical for surgical planning and prognosis. Parameters such as hole dimensions, base and minimum linear diameter (MLD), hole depth, and volume have been shown to be closely related to visual prognosis during preoperative evaluation [3,32]. Furthermore, hole chronicity is a determinant of surgical success and postoperative visual improvement [33]. Accurate segmentation of intraretinal cysts is also crucial for monitoring retinal fluid distribution and assessing treatment response [34,35]. By generating automatic and reproducible segmentation for MHs and cysts, this study lays a solid foundation for the future derivation of reliable and comparable measurements such as MLD, basal diameter (BD), depth, and volume. Furthermore, the degree to which automated measurements can be interpreted under various conditions is also important; this is where SHAP analysis, which converts descriptions into regime-based quantitative signals, comes into play.

In our study, we applied SHAP analysis to understand the model’s decision-making processes and improve its clinical interpretability. SHAP is used in medical image analysis to improve the understandability of black-box models [17,36]. In the OCT literature, SHAP has been used primarily for interpreting of feature/layer thickness-level annotations and classification models [37]. Explainability in the context of macular holes has been mostly reported with Grad-CAM visualizations [10,38]. Such saliency maps need not be limited to images alone; they can be quantified against clinical segmentation and evaluated with standard metrics. Quantitatively comparing the annotation with the clinical mask rather than limiting the annotation to images alone provides a more robust assessment against biases inherent to saliency methods (e.g., edge/contrast sensitivity) [39]. Saporta et al. standardized the quantitative comparison of saliency heatmaps with clinical segmentation masks on chest radiographs and systematically evaluated multiple saliency methods and architectures with metrics such as IoU and pointing-game [40]. In this study, we not only presented SHAP overlay images but also numerically evaluated the overlap of the overlay with the predicted and true masks using APIL, Dice, and Leak metrics. Furthermore, we classified these focal patterns into retinal-dominant, peri-lesion, and narrow-coverage regimes using PCA and k-means-based factor analysis, making the model’s explanations interpretable not only visually but also numerically and categorically. This approach allows for the direct identification of error sources (such as small cyst size, artifacts, and narrow-coverage) associated with the explanations, enabling clinical prediction of cases in which the model can be trusted.

For clinical use, the three annotation patterns can be interpreted as a quality control/warning layer for the model. In the retina-dominant profile, the output is often reassuring because the focus is primarily within the retina and close to the lesion. However, the risk of false positives should be reviewed due to the possibility of a non-specific focus, especially along bright bands and border reflections. The peri-lesion profile indicates a focus pattern that often follows the lesion vicinity and may still coincide with acceptable segmentation quality. In this case, checking for artifacts, segment boundaries, and possible over-segmentation is recommended, and re-acquisition or manual correction should be performed if necessary. The narrow-coverage profile is most often seen in small/fragmented cysts and thick retinal slices. It is appropriate to examine adjacent slices, evaluate the volume in three dimensions, and adjust the threshold/filter settings if necessary. While segmentation masks are spatial outputs, they do not explain why a prediction was produced nor whether it is trustworthy. Therefore, our SHAP analysis is not used to rediscover lesion location but to derive quantitative, case-level reliability signals by measuring attribution–mask alignment and organizing explanations into operational regimes (retina-dominant, peri-lesion, and narrow-coverage). In clinical deployment, where ground truth masks are unavailable, the Pred-referenced regime can serve as a practical self-consistency indicator: peri-lesion and retina-dominant patterns generally coincide with stable Dice/IoU behavior, whereas the narrow-coverage pattern and/or large attribution drift (high COM-dist) flag cases that warrant additional review. Notably, in the retina-dominant regime, the attribution mass is concentrated within retinal tissue and remains spatially close to the lesion region (low COM-dist), but it is not necessarily confined to the lesion mask (often accompanied by high leakage), which motivates treating the regimes as warning/triage signals rather than as guarantees of correctness. Recent Shapley-based studies similarly emphasized that pixel-level heatmaps often require additional interpretation (e.g., grouping analysis) to become actionable [41]. Ren et al. introduced a contrast-level Shapley framework to assess how MRI contrasts contribute to segmentation decisions [41]. In contrast, our objective is not to explain input modalities, but to operationalize pixel-level GradientSHAP into a deployment-oriented reliability layer for identifying potentially unreliable segmentations when clinical ground truth is unavailable.

For both holes and cysts, the retina-dominant cluster was the most common cluster, and the narrow-coverage cluster was the least common. In holes, central slices usually contained a larger lesion area, so narrow-coverage patterns were less frequent and the segmentation boundaries appeared more reliable than in cysts. In cysts, the narrow-coverage cluster was also the least common cluster, but its segmentation reliability was relatively lower because it could be seen even in central slices. However, in current clinical practice, precise margin-related measurements of cysts are not routinely performed. The primary clinical need is cyst detection and monitoring. Therefore, the practical impact is limited.

The correspondence of model foci in macular holes to clinically significant regions is consistent with explainability examples in the literature. The hole edges are frequently highlighted in heatmaps generated by Grad-CAM-like methods. Indeed, in a multicenter closure prediction study by Hu et al., it was shown that the focus was concentrated on the hole and surrounding retina [10]. Similarly, Mariotti et al.’s AI-based quantitative biomarker analysis correlated improvement in ELM/EZ integrity with visual gain, while the images highlighted areas adjacent to the macular hole margin [38]. Because ELM/EZ continuity at the photoreceptor band level is closely linked to visual prognosis, interruptions in these layers become apparent in the annotation maps [38]. In our study, the SHAP overlays also highlighted exposed retinal pigment epithelium (RPE) regions at the base of the MHs, outer plexiform layer borders, and cyst walls. This finding suggests that the model uses regions of transition from hyperreflective to hyporeflective areas as discriminatory cues in OCT decisions; in other words, focus tends to be concentrated at clinically significant borders and interfaces.

This study has several limitations. First, the evaluation was performed with a single data source, and external validation was not performed. Therefore, the generalizability of the findings is limited. We focused on the publicly available OIMHS dataset to ensure full reproducibility (labels, preprocessing, and evaluation) and because annotated OCT volumes for macular hole and cyst segmentation are limited in an open multi-center form; external validation requires access to additional labeled cohorts, which is outside the scope of this initial benchmark study. Second, the 2.5D architecture may be more susceptible to uncertainty, particularly in small/fragmented cysts, as it cannot capture full 3D continuity. Third, SHAP-based quantification is sensitive to the selection of τ thresholds, preprocessing steps, and the need for pixel-based calibration of center-of-mass distance-based measurements. Fourth, label subjectivity in manual masks can also influence the results. Finally, the lack of comprehensive, multicenter comparisons with current 3D/hybrid architectures results in uncertain generalizability and comparability. Fifth, we did not conduct a clinical user study or expert-centered evaluation; therefore, the clinical utility of the proposed explanations has not yet been validated. As future work, we will validate the model across centers/devices and run ablation studies comparing 2.5D with 2D/3D/hybrid architectures. We will quantify the domain shift across devices and centers by reporting performance and calibration per site/scanner and assess robustness via simple adaptation strategies. We will also analyze the sensitivity of SHAP to the baseline choice and τ levels, and assess deployment-oriented improvements such as uncertainty estimation and clinician-in-the-loop verification. In addition, we will perform an expert-in-the-loop evaluation in which ophthalmology specialists assess the segmentation quality and explanation usefulness or trustworthiness using representative cases.

## 5. Conclusions

The proposed model can achieve high accuracy and good calibration in macular hole and cyst segmentation. By introducing SHAP-based coverage metrics and regime labels, annotations are expressed not only visually but also quantitatively and categorically, bringing the clinical decision closer to the data. The model focus was more stable in MHs, whereas coverage loss and leakage were more pronounced in small or fragmented cysts. Overall, the framework adds a quality-control layer to reporting, numerically standardizing decision steps and supporting the routine use of automated segmentation. Future clinical studies with multicenter and multi-device validation, comparisons with different saliency methods, and 3D-weighted architectures are required.

## Figures and Tables

**Table 1:** Layer features of the proposed model.

d1: DoubleConv (3→48) × 512 × 512 $y^{\left(1\right)}={Conv}_{3\times 3}(x)$ $GN(y^{\left(1\right)})=GroupNorm(y^{\left(1\right)})$ $v^{\left(1\right)}=ReLU({GN(y}^{\left(1\right)}))$ $y^{\left(2\right)}={Conv}_{3\times 3}(v^{\left(1\right)})$ $c_{1}=ReLU({GN(y}^{\left(2\right)}))\in R^{B\times 48\times 512\times 512}$ $p_{1}=MaxPool(c_{1})\in R^{B\times 48\times 256\times 256}$	Upsample (bilinear) ve skip-concat1 (c4↑ + c3) × 128 × 128 ${\tilde{c}}_{4}=Bilinear(c_{4})$ $s_{3}=Concat({\tilde{c}}_{4},c_{3})\in R^{B\times (384+192)\times 128\times 128}$ $u_{3}=DC(s_{3})\in R^{B\times 192\times 128\times 128}$
d2: DoubleConv (48→96) × 256 × 256 $c_{2}=DC(p_{1})\in R^{B\times 96\times 256\times 256}$ $p_{2}=MaxPool(c_{2})\in R^{B\times 96\times 128\times 128}$	Upsample ve skip2 (u3↑ + c2) × 256 × 256 ${\tilde{u}}_{3}=Up(u_{3})$ $s_{2}=Concat({\tilde{u}}_{3},c_{2})\in R^{B\times (192+96)\times 256\times 256}$ $u_{2}=DC(s_{2})\in R^{B\times 96\times 256\times 256}$
d3: DoubleConv (96→192) × 128 × 128 $c_{3}=DC(p_{2})\in R^{B\times 192\times 128\times 128}$ $p_{3}=MaxPool(c_{3})\in R^{B\times 192\times 64\times 64}$	Upsample ve skip3 (u2↑ + c1) × 512 × 512 ${\tilde{u}}_{2}=Up(u_{2})$ $s_{1}=Concat({\tilde{u}}_{2},c_{1})\in R^{B\times (96+48)\times 512\times 512}$ $u_{1}=DC(s_{1})\in R^{B\times 48\times 512\times 512}$
$d4\;(\mathrm{Bottleneck}):\;\mathrm{DoubleConv}\;(192\to 384)\;\times \;c_{4}=DC(p_{3})\in R^{B\times 384\times 64\times 64}$	Output: 1 × 1 Conv (48→5) and Softmax

**Table 2:** Hardware and software environment.

Component	Feature
GPU	NVIDIA GeForce RTX 4050 Laptop GPU (6 GB VRAM), CUDA runtime 12.5
CPU	Intel Core i7-13700H (14 core/20 thread)
RAM	15.7 GB
OS	Windows 11 (Build 26100)
Python	3.12.10
PyTorch	2.5.1+cu121 (CUDA build 12.1; 12.5 compatible with the driver)
torchvision	0.20.1+cu121
scikit-learn	1.7.2
Seed	42

**Table 3:** SHAP targets and running parameters.

Setting/Output	Value/Description
Target segments	370 OCT slices containing holes/cysts in the test set
Target classes	hole, cyst
Normalization	z-score (per image and channel)
Attribution method	GradientSHAP
Number of samples	128
Baseline number	16
Baseline type	Gaussian fuzzy noise (σ ≈ 0.8)
Clip (percentile)	0.1–99.9
Tissue restriction	Intraretinal only
τ (top-τ%)	10
Ablation τ	20
Evaluation modes	GT and Pred
Reported metrics	APIL, ARIL, Dice, Leak, COM-dist
Images	Continuous heatmap; binary overlay of top-τ% attribution on lesion mask

**Table 4:** Parameter definitions.

Metrics	Definition
Dice (Dice Coefficient)	Overlap between the predicted and true masks. Reported per class and as a macro average excluding background. Range: 0–1 (higher is better).
IoU (intersection-over-union)	Ratio of the intersection area to the union area between predicted and true masks. Reported per class and macro (BG excluded). Range: 0–1 (higher is better).
HD95 (95th-percentile Hausdorff Distance)	95th percentile of worst point-to-point distances between two boundaries. Unit: pixels. (Lower is better.)
ECE (expected calibration error)	Calibration of predicted probabilities computed as a weighted average of confidence–accuracy gaps across bins. Range: 0–1 (lower is better).
APILτ (Attribution Precision in Lesion)	Proportion of top-τ positive attributions that fall inside the lesion (present-only). ≈precision. Range: 0–1 (higher is better).
ARILτ (Attribution Recall in Lesion)	Proportion of the true lesion that is covered by the top-τ positive attributions. ≈recall. Range: 0–1 (higher is better).
Diceτ (Dice at top-τ attribution)	Dice overlap between the binarized top-τ attribution mask and the lesion mask (present-only). ≈F1-score. Range: 0–1 (higher is better).
Leakτ (leakage at top-τ)	Proportion of top-τ attributions that fall outside the lesion (present-only). ≈1 − precision. Range: 0–1 (lower is better).
COM-dist (center-of-mass distance)	Euclidean distance between attribution and lesion centers of mass. Unit: pixels. (Lower is better.)
nCOM (normalized COM-dist)	COM-dist divided by √ (lesion area) to reduce sensitivity to lesion size. Unit: dimensionless. (Lower is better.)

**Table 5:** Test set and post-processed metrics.

Class	Dice (PP)	IoU (PP)	HD95 (px) *	ECE *
BG	0.990	0.980	-	-
retina	0.984	0.969	-	-
hole	0.941	0.910	7.8	0.007
cyst	0.874	0.805	4.1	0.010
choroid	0.953	0.911	-	-
Mean	0.947	0.915	6.0	0.008

* HD95/ECE averages are calculated only for lesion classes (hole and cyst). Abbreviations—Dice (PP): Dice coefficient (post-processed); IoU (PP): intersection-over-union (post-processed); HD95 (px): 95th-percentile Hausdorff distance; ECE: expected calibration error; BG: background. Note—metrics are macro per-slice averages; therefore, Dice and IoU in the table need not obey the closed-form Dice–IoU relation.

**Table 6:** Class-balanced macro means (present-only).

Mode	APIL10	Dice10	Leak10	APIL20	Dice20	Leak20	COM-Dist (px)
GT	0.077	0.127	0.923	0.039	0.070	0.961	10.24
Pred	0.072	0.123	0.928	0.036	0.067	0.964	8.98

APIL10/20: Attribution Precision in Lesion (τ = 10%/20%); Dice10/20: Dice coefficient (τ = 10%/20%); Leak10/20: leakage (1 − APIL10/20); COM-dist (px): center-of-mass distance (pixels); GT: ground truth-referenced mode; Pred: prediction-referenced mode.

**Table 7:** SHAP factor analysis cluster summaries for Hole.

Cluster	Mode	APIL20	Dice20	Leak20	ARIL20	FP	LP	PAM	FC	COM-Dist
NC (*n* = 0)	GT	-	-	-	-	-	-	-	-	-
PL (*n* = 79)	GT	0.083	0.145	0.917	0.684	−0.21	6487.4	160.4	3.88	16.22
RD (*n* = 77)	GT	0.017	0.032	0.983	0.659	−0.24	1282.4	220.8	3.99	7.84
NC (*n* = 15)	Pred	0.003	0.006	0.997	0.397	−0.63	273.5	484.3	3.73	7.34
PL (*n* = 71)	Pred	0.078	0.139	0.922	0.719	−0.19	5748.1	163.0	3.92	14.38
RD (*n* = 75)	Pred	0.020	0.039	0.980	0.758	−0.18	1409.2	221.1	3.99	6.24

APIL20: Attribution Precision in Lesion at τ = 20; Dice20: Dice coefficient at τ = 20; Leak20 = 1 − APIL20; ARIL20: Attribution Recall in Lesion at τ = 20; FP: Focus Proximity = −nCOM; LP: Lesion Pixels; PAM: Positive Attribution Mass; FC: Focus Concentration = APIL5/APIL20; GT: ground truth-referenced mode; Pred: prediction-referenced mode; NC: narrow-coverage; PL: peri-lesion; RD: retinal-dominant.

**Table 8:** SHAP factor analysis cluster summaries for cyst.

Cluster	Mode	APIL20	Dice20	Leak20	ARIL20	FP	LP	PAM	FC	COM-Dist
NC (*n* = 4)	GT	0.000	0.000	1.000	0.494	−22.6	7.0	69.3	4.00	52.02
PL (*n* = 113)	GT	0.060	0.111	0.940	0.719	−0.16	4386.4	145.2	3.48	10.17
RD (*n* = 253)	GT	0.012	0.024	0.988	0.654	−0.49	930.4	213.5	3.92	6.91
NC (*n* = 14)	Pred	0.001	0.002	0.999	0.568	−1.90	97.2	485.4	3.97	14.21
PL (*n* = 111)	Pred	0.063	0.116	0.937	0.775	−0.16	4244.8	147.9	3.52	9.89
RD (*n* = 232)	Pred	0.014	0.027	0.986	0.763	−0.28	957.5	214.6	3.93	6.75

APIL20: Attribution Precision in Lesion at τ = 20; Dice20: Dice coefficient at τ = 20; Leak20 = 1 − APIL20; ARIL20: Attribution Recall in Lesion at τ = 20; FP: Focus Proximity = −nCOM; LP: Lesion Pixels; PAM: Positive Attribution Mass; FC: Focus Concentration = APIL5/APIL20; GT: ground truth-referenced mode; Pred: prediction-referenced mode; NC: narrow-coverage; PL: peri-lesion; RD: retinal-dominant.

**Table 9:** Comparison with recent studies on macular hole (MH) and intraretinal cyst (IRC) segmentation/measurement on OCT datasets.

Study	Dataset	Target(s)	Method/Backbone	Reported Metric(s)
Proposed model	OIMHS	MH + cyst	2.5D U-Net-48 + GroupNorm	Dice/IoU: hole 0.941/0.910, cyst 0.874/0.805
Herath et al. [9]	OIMHS	MH segmentation	U-Net variants with different CNN/Transformer encoders	- Best: InceptionNetV4 + U-Net Dice = 0.9672 - Close baseline: U-Net Dice = 0.9593 (HD95 = 1.9987) - Other: SegResNet Dice = 0.8830 (HD95 = 1.9274); Transformer + U-Net Dice = 0.78245 (HD95 = 9.7801)
Kulyabin et al. [18]	OIMHS	MH + IRC	SAM 2/MedSAM 2 (prompt-driven volumetric segmentation)	Overall Dice: MH 0.913, IRC 0.902 (MedSAM 2 box selection)
Frawley et al. [19]	Clinical OCT volumes (49 B-scans/volume)	MH	Robust 3D U-Net variants	Mean IoU = 0.876 ± 0.012; per-image Dice = 0.94–0.97 range
Chen et al. [20]	104 consecutive MH	MH morphology	Automated 3D segmentation algorithm	Accuracy = 99.19% (30 eyes); MA–BA center misalignment >150 μm in 70%; inter-observer 95% LoA for MLD −140 to 146 μm
Pereira et al. [21]	Swept-source OCT case series	MH volume	Fully 3D CNN segmentation	Manual vs. automated MH volume: R^2^ = 0.94 (*n* = 24); volume vs. VA change R^2^ = 0.53 vs. MLD R^2^ = 0.39
Girish et al. [22]	OPTIMA OCSC	IRC/cyst	Fully convolutional network	Dice = 0.71, Recall = 0.66, Precision = 0.79
Ganjee et al. [23]	OPTIMA + KERMANY	IRC/cyst	Generalizable U-Net-based approach	Mean Dice: 0.78 (OPTIMA) and 0.81 (KERMANY)
Gopinath & Sivaswamy [24]	OCSC + DME + AEI	IRC/cyst	Selective enhancement + CNN + clustering	Proposed + K-means Dice: 0.69 (OCSC); 0.67 (DME); 0.79 (AEI)

OIMHS, Optical Coherence Tomography Image Dataset Based on Macular Hole Manual Segmentation; OCT, optical coherence tomography; MH, macular hole; IRC, intraretinal cyst(s); U-Net, U-shaped convolutional neural network; CNN, convolutional neural network; GroupNorm, Group Normalization; IoU, Intersection-over-Union; HD95, 95th-percentile Hausdorff distance; SegResNet, segmentation residual network; LoA, limits of agreement; MLD, minimum linear diameter; R^2^, coefficient of determination; OPTIMA, OPTIMA OCT dataset/challenge; OCSC, OPTIMA Cyst Segmentation Challenge; DME, diabetic macular edema.

## Data Availability

The data presented in this study are available on request from the corresponding author due to data privacy.

## Supplementary Materials

The following supporting information can be downloaded at: https://www.mdpi.com/article/10.3390/diagnostics16010097/s1, Table S1. Extended performance and computational profiling of the proposed UNet-48 + GN (2.5D) and classic U-Net-64 + BN baselines (2.5D and 2D) on OIMHS at full resolution (512 × 512); Table S2. τ sensitivity (GT-referenced); Table S3. τ sensitivity (Pred-referenced); Table S4. COM-dist summary; Table S5. Segmentation performance by SHAP regime for Macular Hole; Table S6. Segmentation performance by SHAP regime for Intraretinal Cyst.
