# Supplementary material for: Quantifying Explainability in OCT Segmentation of Macular Holes and Cysts: A SHAP-Based Coverage and Factor Contribution Analysis
Source: Diagnostics (Basel). 2025 Dec 27;16(1):97. doi: 10.3390/diagnostics16010097 (PMC12785673; doi:10.3390/diagnostics16010097)
Supplement: Supplementary file 1 [file diagnostics-16-00097-s001.zip › diagnostics-3951924-supplementary.pdf]

1. Extended Baseline Comparison and Computational Profiling

Supplementary Table S1 summarizes segmentation performance and computational characteristics of the proposed 2.5D UNet-48 + Group Normalization (GN) and two classic U-Net baselines (UNet-64 + Batch Normalization, BN) evaluated at full resolution (512×512) on the OIMHS test set. Performance is reported as Dice/IoU for the mean over all classes (including background) and for the two target biomarkers (macular hole and intraretinal cyst). Computational profiling includes the number of trainable parameters and empirical inference/training peak GPU memory and latency measured on the target GPU under mixed precision (AMP). Overall, the table provides a compact view of accuracy–efficiency trade-offs across 2D and 2.5D settings under the same preprocessing and evaluation pipeline.

Overall, the proposed UNet-48+GN provides a favorable balance between accuracy and computational footprint: it achieves the highest mean and lesion Dice/IoU (e.g., Mean Dice/IoU 0.947/0.915; MH 0.941/0.910; cyst 0.874/0.805) while using substantially fewer parameters than the UNet-64 backbones (4.383M vs ~7.789M). Compared with the classic 2.5D UNet-64+BN, UNet-48+GN offers markedly improved macular hole performance and higher overall accuracy under comparable runtime/memory constraints, supporting its selection as a more memory-efficient 2.5D backbone on limited hardware. Although the 2D UNet-64 baseline is faster, it shows lower overall segmentation quality than the proposed model, indicating that the proposed 2.5D configuration yields a better accuracy–efficiency trade-off for the targeted biomarkers.

Supplementary Table S1. Extended performance and computational profiling of the proposed UNet-48+GN (2.5D) and classic U-Net-64+BN baselines (2.5D and 2D) on OIMHS at full resolution (512×512). Mean metrics include BG. Inference/train-step measurements are obtained on the target GPU under AMP.

**Metric**	**UNet-48 + GN (2.5D, C=3)**	**UNet-64 + BN (2.5D, C=3)**	**UNet-64 + BN (2D, C=1)**
Params (M)	4.383	7.789	7.788
Inference (ms/img)	1204.92	864.53	51.04
Inference peak mem (GB)	0.447	0.548	1.296
Train-step peak mem (GB)	1.135	0.956	1.705
Mean (all classes) Dice / IoU	0.947 / 0.915	0.877 / 0.836	0.920 / 0.874
MH (hole) Dice / IoU	0.941 / 0.910	0.709 / 0.708	0.917 / 0.883
Cyst Dice / IoU	0.874 / 0.805	0.852 / 0.776	0.843 / 0.771
2D, two-dimensional; 2.5D, two-and-a-half-dimensional (stacked adjacent slices); C, number of input channels/slices; U-Net, U-shaped convolutional neural network; UNet-48/UNet-64, U-Net with base channel width 48/64; GN, Group Normalization; BN, Batch Normalization; Params (M), number of trainable parameters in millions; ms/img, milliseconds per image; peak mem, peak GPU memory; GB,			

gigabytes; Dice, Dice similarity coefficient; IoU, Intersection-over-Union (Jaccard index); MH, macular hole.

2. Sensitivity analysis for τ thresholds (top-% SHAP masks)

As τ increases from 5% to 20%, APIL decreases and Leakage increases as expected (wider attribution coverage), while ARIL remains relatively stable, indicating that conclusions are not driven by a single τ choice (Supplementary Table S2).

Supplementary Table S2. τ sensitivity (GT-referenced)

Class	τ (top-%)	n	APIL	ARIL	Diceτ	Leak
Hole	5%	156	0.1938	0.660	0.266	0.806
Hole	10%	156	0.1006	0.671	0.162	0.899
Hole	20%	156	0.0503	0.671	0.089	0.950
Cyst	5%	370	0.0962	0.636	0.153	0.904
Cyst	10%	370	0.0526	0.668	0.093	0.947
Cyst	20%	370	0.0267	0.672	0.050	0.973

τ (top-%): percentage threshold used to binarize the SHAP attribution map by selecting the top-τ% pixels; APIL: Attribution Precision in Lesion; ARIL: Attribution Recall in Lesion; Diceτ: Dice at top-τ attribution; Leak: leakage at top-τ; GT: Ground truth; n: number of evaluated slices/samples.

The same monotonic τ trend is observed under Pred-referenced evaluation. Overall patterns and class-wise interpretations remain consistent across τ (Supplementary Table S3).

Supplementary Table S3. τ sensitivity (Pred-referenced)

Class	τ (top-%)	n	APIL	ARIL	Diceτ	Leak
Hole	5%	161	0.1724	0.700	0.249	0.828
Hole	10%	161	0.0879	0.707	0.146	0.912
Hole	20%	161	0.0440	0.707	0.080	0.956
Cyst	5%	357	0.1042	0.722	0.166	0.896
Cyst	10%	357	0.0566	0.755	0.100	0.943
Cyst	20%	357	0.0287	0.759	0.054	0.971

τ (top-%): percentage threshold used to binarize the SHAP attribution map by selecting the top-τ% pixels; APIL: Attribution Precision in Lesion; ARIL: Attribution Recall in Lesion; Diceτ: Dice at top-τ attribution; Leak: leakage; Pred: prediction; n: number of evaluated slices/samples.

COM-dist is threshold-independent in our summary outputs and provides complementary evidence about attribution localization consistency between GT- and Pred-referenced modes(Supplementary Table S4).

Supplementary Table S4. COM-dist summary

Class	COM-dist mean (GT-ref)	COM-dist mean (Pred-ref)
Hole	12.084	9.929

Cyst	8.391	8.020
COM-dist: Euclidean distance between the center-of-mass of the positive SHAP attribution mass and the center-of-mass of the lesion mask (pixels); GT-ref / Pred-ref: SHAP metrics computed with respect to the ground-truth lesion mask (GT) or the predicted lesion mask (Pred).		

3. Segmentation Performance Stratified by SHAP Regimes

Total N denotes the number of slices assigned to each SHAP regime. Valid N counts slices with non-zero Dice and IoU (Dice>0 and IoU>0), reported to avoid trivial zero-overlap cases dominating summary statistics. Results are reported for both GT-referenced (GT) and Pred-referenced (Pred) modes. In general, peri-lesion and retina-dominant regimes show stable overlap scores, while narrow-coverage occurs rarely for hole in GT mode (Total N = 0 in this cohort) (Supplementary Table S5).

Supplementary Table S5. Segmentation performance by SHAP regime for Macular Hole.

**Regime**	**Mode**	**Total N**	**Valid N (Dice>0 & IoU>0)**	**Dice (mean ± std)**	**IoU (mean ± std)**
Peri-lesion	GT	79	28	0.842 ± 0.097	0.737 ± 0.130
Retina-dominant	GT	77	26	0.831 ± 0.167	0.736 ± 0.169
Narrow-coverage	GT	0	–	–	–
Peri-lesion	Pred	71	27	0.847 ± 0.091	0.744 ± 0.122
Retina-dominant	Pred	75	27	0.816 ± 0.167	0.715 ± 0.173
Narrow-coverage	Pred	15	4	0.908 ± 0.039	0.833 ± 0.066
Total N is the number of slices assigned to each SHAP regime; valid N counts slices with non-zero Dice and IoU; IoU denotes Intersection over Union.					

Total N denotes the number of slices assigned to each SHAP regime. Valid N counts slices with non-zero Dice and IoU (Dice>0 and IoU>0), reported to reduce the influence of empty/degenerate overlap cases. Results are reported for both GT-referenced (GT) and Pred-referenced (Pred) modes. Peri-lesion typically exhibits the highest overlap consistency, whereas narrow-coverage corresponds to a small subset of challenging cases with reduced lesion coverage (small Total N), motivating its use as a “review-needed” indicator rather than a guarantee of segmentation quality (Supplementary Table S6).

Supplementary Table S6. Segmentation performance by SHAP regime for Intraretinal Cyst.

**Regime**	**Mode**	**Total N**	**Valid N (Dice>0 & IoU>0)**	**Dice (mean ± std)**	**IoU (mean ± std)**
Narrow-coverage	GT	4	3	0.776 ± 0.127	0.647 ± 0.162
Peri-lesion	GT	113	86	0.840 ± 0.151	0.747 ± 0.162

Retina-dominant	GT	253	165	0.805 ± 0.206	0.714 ± 0.202
Narrow-coverage	Pred	14	10	0.878 ± 0.077	0.790 ± 0.111
Peri-lesion	Pred	111	85	0.842 ± 0.152	0.749 ± 0.163
Retina-dominant	Pred	232	151	0.801 ± 0.207	0.708 ± 0.203

Total N is the number of slices assigned to each SHAP regime; valid N counts slices with non-zero Dice and IoU; IoU denotes Intersection over Union.
